# Neurological Conundrum: A Case of Cerebellar Hemispheric Enlargement and Atypical Symptoms

**DOI:** 10.7759/cureus.58096

**Published:** 2024-04-12

**Authors:** Paschyanti R Kasat, Pratap Parihar, Shivali V Kashikar, Pratiksha Sachani, Bhagyasri Nunna

**Affiliations:** 1 Radiodiagnosis, Jawaharlal Nehru Medical College, Datta Meghe Institute of Higher Education & Research, Wardha, IND

**Keywords:** differential diagnosis, management strategies, magnetic resonance imaging, diagnostic challenges, atypical neurological symptoms, cerebellar hemispheric enlargement

## Abstract

Cerebellar hemispheric enlargement with atypical neurological symptoms poses diagnostic challenges in clinical practice. We present the case of a 57-year-old female with persistent headache, left facial paraesthesia, dysarthria, gait ataxia, and longstanding neck swelling. Imaging studies revealed enlargement of the left cerebellar hemisphere with associated mass effect and compression of adjacent structures. The underlying etiology remained uncertain despite extensive evaluation, including magnetic resonance imaging and angiography. Differential diagnoses included neoplastic, vascular, inflammatory, and metabolic etiologies, but none fully accounted for the clinical findings. Management strategies focused on symptomatic relief and close monitoring. This case underscores the complexity of diagnosing and managing patients with rare neurological manifestations and highlights the need for continued research and collaborative approaches in optimising patient care.

## Introduction

Cerebellar hemispheric enlargement is a rare neurological finding characterised by abnormal enlargement of one or both cerebellar hemispheres. This condition can present with various symptoms, including headache, ataxia, dysarthria, and cranial nerve deficits, depending on the extent and location of the enlargement. The underlying etiology of cerebellar hemispheric enlargement may include neoplastic, vascular, inflammatory, infectious, or metabolic causes [1]. One of the potential etiologies of cerebellar hemispheric enlargement is a cerebellar astrocytoma, a primary central nervous system tumour arising from astrocytes within the cerebellum. Astrocytomas represent the most common type of primary brain tumour in children, with pilocytic astrocytoma being the most common histological subtype [2]. However, astrocytomas can also occur in adults, albeit less frequently, and may present with symptoms related to mass effects, such as headache, ataxia, and cranial nerve deficits [3].

Another potential cause of cerebellar hemispheric enlargement is vascular malformation, including arteriovenous malformations (AVMs) or developmental venous anomalies (DVAs). AVMs are abnormal tangles of blood vessels that bypass normal brain tissue, leading to abnormal blood flow and potential haemorrhage [4]. DVAs, on the other hand, are benign vascular variants characterised by dilated venous structures without arteriovenous shunting [5]. Both AVMs and DVAs can cause mass effects on surrounding brain tissue, leading to neurological symptoms such as headache, ataxia, and cranial nerve deficits. Inflammatory and infectious etiologies, such as multiple sclerosis (MS) or neurocysticercosis, should also be considered in the differential diagnosis of cerebellar hemispheric enlargement. MS is an autoimmune inflammatory disorder characterised by demyelination and axonal damage within the central nervous system, which can lead to various neurological symptoms, including ataxia and dysarthria [6]. Neurocysticercosis, on the other hand, is a parasitic infection caused by the larval stage of the pork tapeworm *Taenia solium*, which can lead to the formation of cystic lesions within the brain parenchyma, including the cerebellum [7].

Metabolic disorders, such as mitochondrial diseases or lysosomal storage disorders, may also present with cerebellar hemispheric enlargement as part of their clinical spectrum. Mitochondrial diseases are a heterogeneous group of genetic disorders characterised by mitochondria dysfunction, the cellular organelles responsible for energy production [8]. On the other hand, lysosomal storage disorders are inherited metabolic disorders characterised by abnormal accumulation of various substances within lysosomes, leading to cellular dysfunction and tissue damage [9]. Both mitochondrial diseases and lysosomal storage disorders can affect the central nervous system, leading to neurological symptoms such as ataxia, dysarthria, and cognitive impairment.

## Case presentation

A 57-year-old female presented to our neurology clinic with a chief complaint of persistent headache accompanied by paraesthesia of the left side of her face, dysarthria, and gait ataxia. Additionally, she reported a longstanding swelling over the right side of her neck, which had been present for approximately one year. The patient denied any history of ear-related symptoms, nasal issues, or previous trauma. Her medical history was significant for systemic hypertension. Upon clinical examination, the patient exhibited signs consistent with cerebellar dysfunction, including dysarthria and gait ataxia. A neurological examination revealed no focal deficits besides the observed paraesthesia on the left side of her face. The swelling over the right side of her neck was noted, but examination findings did not suggest an acute infection or inflammation.

**Figure 1:**
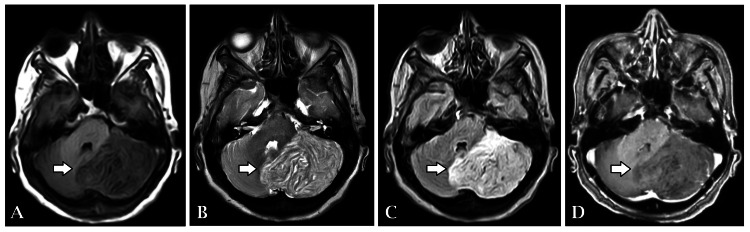
(A, B, C) The left cerebellar hemisphere was enlarged with altered signal intensity, presenting a tigroid appearance on T1-weighted, T2-weighted, and FLAIR images. (D) There was no evidence of enhancement post-contrast FLAIR: fluid-attenuated inversion recovery

**Figure 2:**
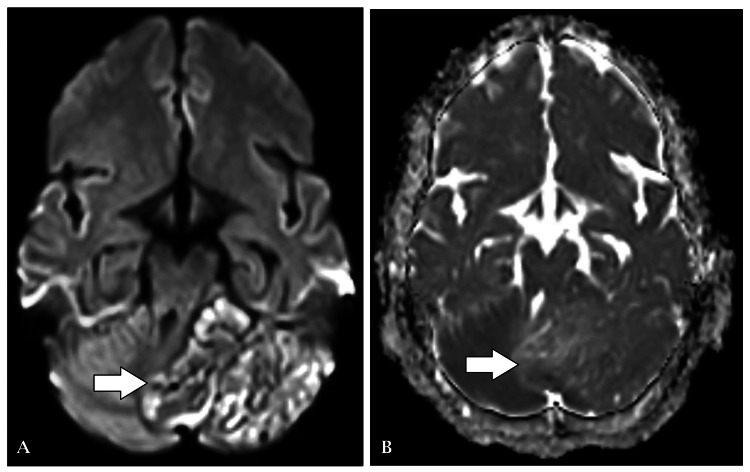
(A) DWI: few areas show restriction. (B) ADC shows a corresponding low signal DWI: diffusion-weighted imaging; ADC: apparent diffusion coefficient

Given the complexity of her symptoms, further diagnostic evaluation was pursued. Magnetic resonance imaging (MRI) of the brain revealed several significant findings. Notably, the left cerebellar hemisphere was enlarged with altered signal intensity, presenting a tigroid appearance on T1-weighted and T2-weighted images. Despite the enlargement, there was no evidence of enhancement post-contrast (Figure 1), and diffusion-weighted imaging (DWI) ruled out infarction (Figure 2).

**Figure 3:**
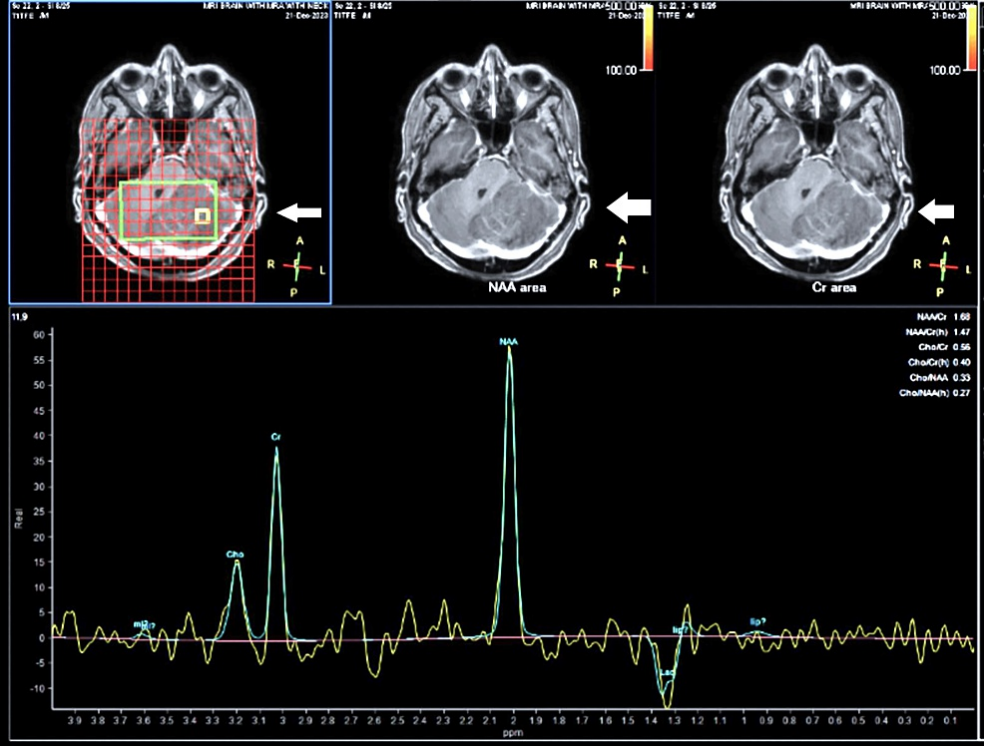
On MR spectroscopy, the choline/creatine ratio is reduced MR: magnetic resonance

The cerebellar enlargement was found to cause a mass effect, resulting in the effacement of the fourth ventricle, compression of adjacent structures such as the midbrain, pons, and medulla, and cerebellar tonsillar herniation with mild occlusive hydrocephalus. Additionally, magnetic resonance spectroscopy revealed metabolic alterations in the affected area, while bilateral white matter hyperintensities on T2-weighted imaging and fluid-attenuated inversion recovery (FLAIR) sequences evidenced small vessel ischemic disease (Figure 3).

Based on the clinical presentation and imaging findings, a diagnosis of cerebellar hemispheric enlargement with associated mass effect and neurological symptoms was established. The underlying etiology remained uncertain, and further investigations were warranted to elucidate the cause of the cerebellar abnormalities and their relation to the patient's neck swelling.

## Discussion

Cerebellar hemispheric enlargement is a rare finding with diverse etiologies, and its association with atypical neurological symptoms presents diagnostic challenges [10]. In this case, the patient presented with persistent headache, left facial paraesthesia, dysarthria, gait ataxia, and a longstanding neck swelling, prompting further investigation. The imaging studies revealed enlargement of the left cerebellar hemisphere with associated mass effect and compression of adjacent structures, complicating the diagnostic process.

One of the primary differential diagnoses for cerebellar hemispheric enlargement is neoplastic etiology, including tumours such as hemangioblastoma, metastases, or primary cerebellar neoplasms [11]. However, in this case, the absence of enhancement post-contrast and DWI ruling out infarction argued against a neoplastic or ischemic etiology. Nonetheless, it is essential to consider the possibility of low-grade tumours or atypical presentations of neoplastic processes, warranting close follow-up and repeat imaging studies.

Vascular anomalies represent another important consideration in patients with cerebellar hemispheric enlargement. In this case, magnetic resonance angiography demonstrated a fetal origin of the left posterior cerebral artery, considered a standard variant [12]. While this finding was not directly related to cerebellar abnormalities, it underscores the importance of evaluating cerebrovascular anatomy in patients with neurological symptoms. Further investigations may include evaluation for arteriovenous malformations, aneurysms, or venous abnormalities, which can contribute to mass effects and neurological deficits.

Inflammatory and infectious etiologies, such as autoimmune cerebellitis or chronic infections, should also be considered in the differential diagnosis. However, in this case, the absence of acute inflammatory markers and clinical findings suggestive of infection argued against these etiologies. Nonetheless, autoimmune processes may present with atypical symptoms and require comprehensive serological and cerebrospinal fluid (CSF) analysis for diagnosis [13].

Metabolic and toxic etiologies represent less common causes of cerebellar hemispheric enlargement but should be considered, particularly in patients with systemic symptoms or a history of exposure to neurotoxic substances. Given the patient's medical history of systemic hypertension, metabolic derangements such as hypertensive encephalopathy or secondary cerebellar changes due to vascular pathology should be considered [14]. However, the absence of acute hypertensive crisis or typical radiological findings argued against this etiology in our case.

The management of cerebellar hemispheric enlargement depends on the underlying etiology and may include medical therapy, surgical intervention, or conservative management. Close monitoring and symptom management are essential to optimise patient outcomes when the etiology remains uncertain. Long-term follow-up with serial imaging studies is warranted to assess disease progression and response to treatment.

## Conclusions

The case presented herein underscores the complexity inherent in diagnosing cerebellar hemispheric enlargement with atypical symptoms. Despite extensive clinical evaluation and imaging studies, the underlying etiology remained elusive. Differential diagnoses, including neoplastic, vascular, inflammatory, and metabolic etiologies, were considered, but none fully accounted for the patient's presentation. The absence of typical radiological findings and acute inflammatory markers further complicated the diagnostic process. Given the underlying pathology's uncertainty, management strategies were directed towards symptomatic relief and close monitoring. This case highlights the need for continued research and collaborative approaches in managing rare neurological manifestations, emphasising the importance of comprehensive evaluation and long-term follow-up in optimising patient outcomes. Further studies are warranted to elucidate the pathophysiological mechanisms underlying cerebellar abnormalities and their relation to atypical clinical presentations.
